# *LGR5* expression is associated with prognosis in poorly differentiated gastric adenocarcinoma

**DOI:** 10.1186/s12885-021-07913-6

**Published:** 2021-03-06

**Authors:** Takehito Ehara, Takeshi Uehara, Tomoyuki Nakajima, Yasuhiro Kinugawa, Shota Kobayashi, Mai Iwaya, Hiroyoshi Ota, Yuji Soejima

**Affiliations:** 1 Department of Surgery, Shinshu University School of Medicine, Matsumoto, Japan; 2 Department of Laboratory Medicine, Shinshu University School of Medicine, 3-1-1 Asahi, Matsumoto, 390-8621 Japan; 3 Department of Biomedical Laboratory Medicine, Shinshu University School of Medicine, Matsumoto, Japan

**Keywords:** Leucine-rich repeat-containing G-protein-coupled receptor 5, Poorly differentiated gastric adenocarcinoma, RNA in situ hybridization

## Abstract

**Background:**

Leucine-rich repeat-containing G-protein-coupled receptor 5 (LGR5*)* is an important cancer stem cell marker in gastric cancer. However, no detailed studies are available on *LGR5* expression in poorly differentiated gastric adenocarcinoma (PD-AC). Therefore, we investigated the relationship between *LGR5* expression and clinicopathological data in PD-AC.

**Methods:**

*LGR5* mRNA expression levels were quantified in 41 PD-AC specimens using a highly sensitive RNAscope in situ hybridization technique. Epstein–Barr virus (EBV) infection was also detected by EBV in situ hybridization.

**Results:**

*LGR5* expression levels were measured in 38 of 41 PD-AC cases, and 17 cases were identified as *LGR5* high. The frequency of EBV positivity tended to be higher in the *LGR5*-low group than in the *LGR5*-high group (*P* = 0.0764). Furthermore, the frequency of vascular invasion tended to be higher in the *LGR5*-high group than in the *LGR5*-low group (*P* = 0.0764). The overall survival of PD-AC patients in the *LGR5*-high group was significantly lower than in the *LGR5*-low group (log-rank test, *P* = 0.0108). The Cox proportional hazard regression model revealed that the *LGR5*-low group (HR = 0.29; 95% CI: 0.11–0.74; *P* = 0.01) showed independently better OS for PD-AC.

**Conclusions:**

Quantifying the levels of LGR5 expression may facilitate defining prognosis in Japanese patients with PD-AC. Further study of LGR5 in this context is warranted.

## Background

Although the incidence of gastric cancer is decreasing, many individuals develop the disease. Gastric cancer is the fifth most frequent cancer and the third leading cause of cancer death [1]. Various treatment methods for gastric cancer, including surgery, radiation, and chemotherapy, have been performed. Among them, many recent studies have been conducted on cancer stem cells (CSCs), which are present in tumor tissues, and anti-CSC therapies have shown promising results [2]. CSCs are resistant to radiation and chemotherapy, and residual CSCs contribute to tumor regrowth. Therefore, effective anti-CSC therapy should be administered in combination with existing methods, such as chemotherapy and radiotherapy [3]. Some reports have investigated gastric CSCs, which typically express CD44, CD133, and Musashi-1 [4]. Additionally, leucine-rich repeat-containing G-protein-coupled receptor 5 (*LGR5*) was identified as a robust CSC marker in murine gastric cancer [5, 6].

The standard treatment for stage I gastric cancer is curative resection only, and it is associated with an excellent prognosis [7]. On the other hand, the standard treatment for stage II/III gastric cancer is curative resection and adjuvant chemotherapy, but the frequency of recurrence is high [8]. Therefore, it is necessary to improve the prognosis of stage II/III gastric cancer patients. Furthermore, the components of poorly differentiated cancer tissues also have a significant impact on prognosis. In gastric cancer, poorly differentiated adenocarcinoma (PD-AC) has a poor prognosis [9], but the underlying mechanisms remain unclear. Additionally, the expression of CSC markers in poorly differentiated gastric cancer, especially *LGR5*, has not been reported. Therefore, we investigated the clinicopathological relationship between *LGR5* marker expression and prognosis in stage II/III gastric cancer patients.

## Methods

### Patients and materials

We identified 91 PD-AC cases who underwent surgical resection between 2008 and 2018 at six institutes [Shinshu University Hospital (Matsumoto, Japan), Nagano Municipal Hospital (Nagano, Japan), Aizawa Hospital (Matsumoto, Japan), Showa Inan General Hospital (Komagane, Japan), Iida Municipal Hospital (Iida, Japan), and Nagano Matsushiro General Hospital (Nagano, Japan)] and evaluated their clinicopathological features. Of these patients, stage II and III cases were selected. A re-evaluation by two pathologists (T.U. and H.O.) before analysis excluded five cases that did not contain poorly differentiated components, and 41 cases remained as candidates for analysis. This study was approved by the Ethics Committee of Shinshu University, Japan (no. 4088).

### Histopathology, immunohistochemical staining, and evaluation

Paraffin blocks fixed with 8% formaldehyde containing sufficient tumor for analysis were prepared for hematoxylin and eosin (HE) staining and tissue microarray (TMA) analysis by extracting a core with a diameter of 3 mm from each case. Additionally, the TMA was subjected to immunostaining using antibodies against the following mismatch repair proteins (MMRPs): MLH1 (ES05; mouse monoclonal; dilution 1:50), PMS2 (EP51; rabbit monoclonal; dilution 1:40), MSH2 (FE11; mouse monoclonal; dilution 1:50), or MSH6 (EP49; rabbit monoclonal; dilution 1:50; Agilent Technologies, Santa Clara, CA, USA), as described previously [10]. Representative images of the slides were captured with an Olympus DP74 camera (lens, × 40; Olympus, Tokyo, Japan) using the CellSens Standard software (Olympus, Tokyo, Japan). The images were acquired at a resolution of 96 dpi and Adobe Photoshop was used to enhance the resolution of the images to 300 dpi. As reported in our previous paper, the staining results were scored as positive when a nuclear staining pattern was observed. If at least one of the four antibodies did not show expression, MMR protein deficiency was indicated. In the tumor, the tumor-infiltrating lymphocyte (TIL) score was assessed using a four-tier scale and recorded as follows: none: 0; mild: 1; moderate: 2; and marked: 3 [11]. The TIL score was classified as low grade (scores 0 and 1) and high grade (2 and 3).

### EBER in situ hybridization

The EBER in situ hybridization assay was performed on TMA block sections. Epstein–Barr virus (EBV) was identified using EBER probes (ISH iVIEW Blue detection kit; Ventana Medical Systems Inc., Oro Valley, AZ, USA).

### *LGR5* RNA in situ hybridization

*LGR5* mRNA detection was performed using the RNAscope® kit (Advanced Cell Diagnostics, Hayward, CA, USA), as described previously [10]. Additionally, a four-step evaluation method we reported previously was used [10]. Furthermore, *LGR5* mRNA expression levels were categorized as low expression (grades 0, 1+, 2+, and 3+) and high expression (4+). We analyzed the relationship between *LGR5* expression levels and the clinicopathological data and prognosis in patients with PD-AC, particularly regarding overall survival (OS).

### TCGA analysis

mRNA-seq analysis was performed using the TCGA database. The TCGA data were downloaded from cBioPortal (http://www.cbiopor- tal.org/) in the form of mRNA median values. All clinical and pathological data for the TCGA cohort were downloaded from cBioPortal. The TCGA cohort was classified into high or low *LGR5* mRNA expression groups. *LGR5* high indicates *LGR5* mRNA expression ≥ the median, and *LGR5* low indicates *LGR5* mRNA expression < the median. Stage II/III patients were included in the first analysis, and then only Stage II/III patients with histological grade 3 were included in the second analysis.

### Statistical analysis

The chi-squared test was applied to assess statistical significance. A *P*-value < 0.05 was considered to be statistically significant. The OS rates of PD-AC patients were calculated using the Kaplan–Meier method, and differences were compared using the log-rank test. Univariate and multivariate analyses for prognostic factors were performed using the Cox proportional hazard regression model. A *P*-value < 0.05 was considered to be statistically significant. Statistical analysis was performed using JMP version 13 (SAS Institute Japan, Tokyo, Japan).

## Results

### *LGR5* expression in PD-AC

**Fig. 1:**
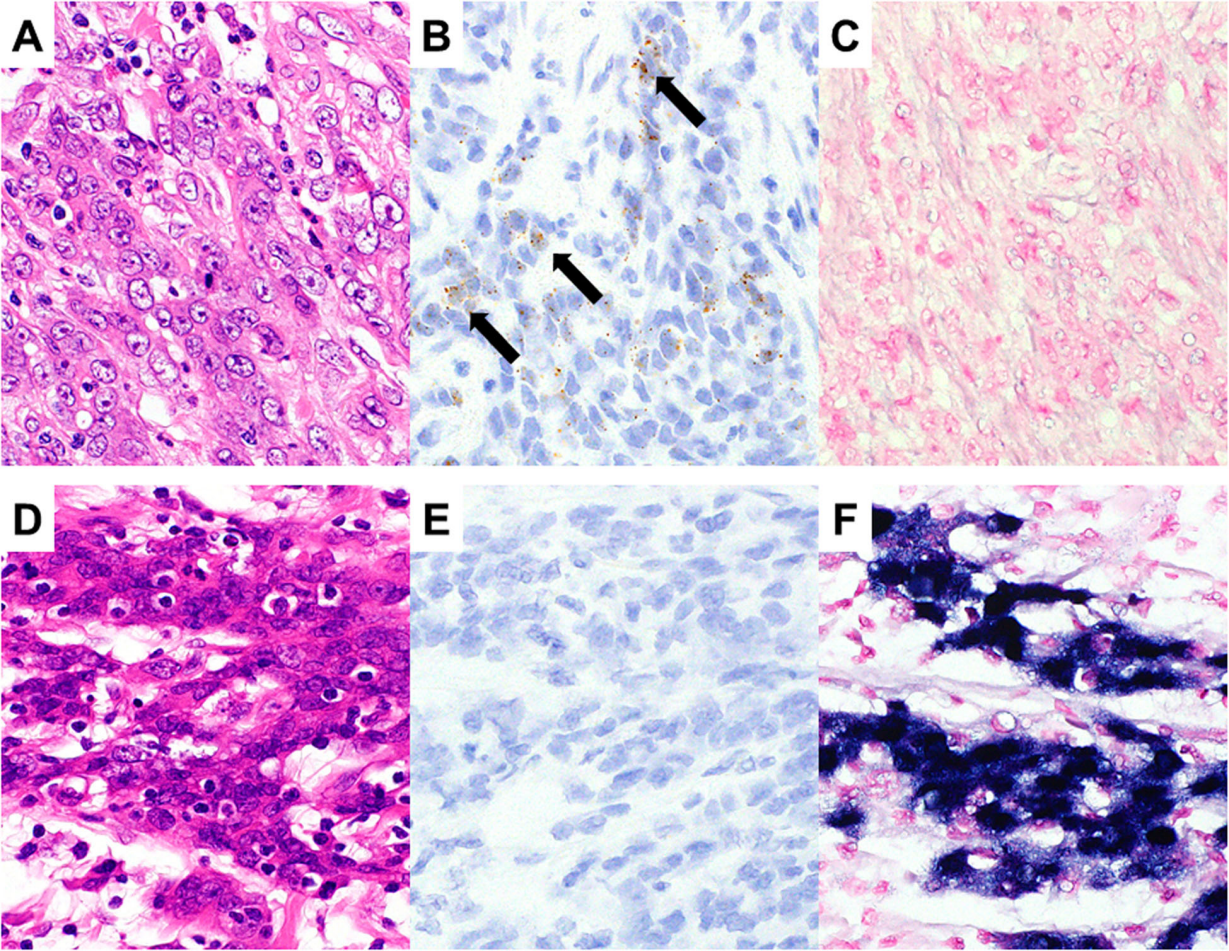
Representative images of *LGR5* and EBV. Representative features in *LGR5*-high expression (**a** and **b**) and *LGR5*-low expression (**d** and **e**) cases. *LGR5*-high expression cases show EBV negativity (**c**). *LGR5*-low expression cases show EBV positivity (**f**). **a** and **d**, HE; **b** and **e**, *LGR5*; **c** and **f**, EBV

In PD-AC patients, 38 of 41 cases showed *LGR5* expression. Among them, 17 cases were identified as *LGR5* high (Fig. 1a and b). Moreover, *LGR5* expression was completely absent in three cases (Fig. 1d and e). *LGR5* expression varied from diffuse to scattered.

### Relationship between *LGR5* expression and clinicopathological characteristics

EBER expression was negative in most cases (Fig. 1c). Although there were few EBV-positive PD-AC cases (Fig. 1f), all exhibited low *LGR5* expression. *LGR5* expression and clinicopathologic data are shown in Table 1. The number of EBV-positive PD-AC cases tended to be higher in the *LGR5*-low expression group than in the *LGR5*-high expression group (*P* = 0.0764). The frequency of vascular invasion tended to be higher in the *LGR5*-high expression group than in the *LGR5*-low expression group (*P* = 0.0764). No significant difference was found between the *LGR5*-high expression group and *LGR5*-low expression group regarding TILs, MSI, histological subtype, or TNM stage.

**Table 1:** *LGR5* expression and clinicopathological characteristics in PD-AC

Factors	*LGR5* expression			
	n	High (*n* = 17)	Low (*n* = 24)	*P*-value
Age				0.0472
> 74 years	19	11	8	
≤ 74 years	22	6	16	
Sex				0.0498
Male	24	13	11	
Female	17	4	13	
EBV				0.0764
Positive	4	0	4	
Negative	37	17	20	
Vascular invasion				0.0764
Present	37	17	20	
Absent	4	0	4	
TIL				0.283
High	25	9	16	
Low	16	8	8	
MSI				0.2176
Present	19	10	9	
Absent	21	7	14	
Differentiated-type				0.2921
Solid-type 1	8	2	6	
Non-solid-type 2	33	15	18	
TNM stage				0.9382
II	19	8	11	
III	22	9	13	

### Prognostic value of *LGR5* in PD-AC

**Fig. 2:**
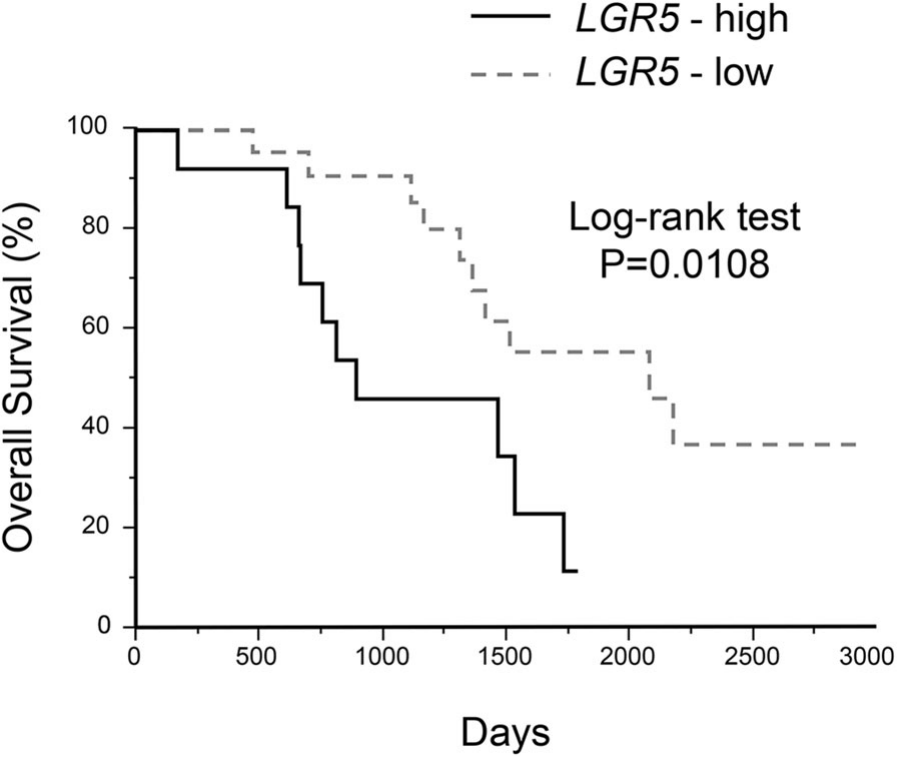
Prognostic value of *LGR5* in PD-AC by Kaplan–Meier analysis. A significant difference was found in OS between PD-AC cases in the *LGR5*-high expression group [median OS; 756 (range; 154.5–1306.5) days] and *LGR5*-low expression [median OS; 1338 (range; 922.75–2022.75) days; log-rank test, *P* = 0.0108]

The prognostic value of *LGR5* expression in PD-AC was analyzed by the Kaplan–Meier method and log-rank test (Fig. 2). The median OS for the study patients was 1146 (range; 635.5–1718) days. A significant difference was found in OS between PD-AC cases in the *LGR5*-high expression group [median OS: 756 (range; 154.5–1306.5) days] and *LGR5*-low expression group [median OS: 1338 (range; 922.75–2022.75) days] (log-rank test, *P* = 0.0108).

We also compared the first quartile with grades 0–2 and the fourth quartile with grade 4. A significant difference was found in OS between PD-AC cases in the first and fourth quartiles (log-rank test, *P* = 0.0072).

We evaluated the relationship between clinicopathological factors and *LGR5* expression regarding OS using a Cox proportional hazard regression model (Table 2), which revealed that the *LGR5*-low expression group (HR = 0.29; 95% CI: 0.11–0.74; *P* = 0.01) had independently better OS for PD-AC.

**Table 2:** Univariate and multivariate analyses of prognostic factors for PD-AC

Factors	Univariate analysis		Multivariate analysis	
	HR (95% CI)	*P*-value	HR (95% CI)	*P*-value
Age: > 74 years vs ≤ 74 years	1.49 (0.57–3.69)	0.3986		
Sex: Male vs Female	0.65 (0.26–1.69)	0.3596		
Vascular invasion: Present vs Absent	3.07 (0.64–55.22)	0.1936		
TIL: Low vs High	1.94 (0.77–4.86)	0.1575		
MSI: Present vs Absent	2.07 (0.79–5.39)	0.1338		
TNM stage: II vs III	0.39 (0.15–0.97)	0.0425	0.36 (0.13–0.89)	0.026
*LGR5*: High vs Low	3.18 (1.24–8.39)	0.0161	3.48 (1.36–9.22)	0.01

### TCGA data analysis

First, we examined the *LGR5* mRNA expression levels in the TCGA cohort of stage II/III patients. There was no significant difference in OS between the *LGR5* high and low groups (log-rank test, *P* = 0.7175). Univariate and multivariate analyses for prognostic factors were performed using the Cox proportional hazard regression model. However, there was no significant difference in the univariate analysis of the TCGA cohort (HR = 0.93; 95% CI: 0.64–1.35; *P* = 0.72).

We then examined the *LGR5* mRNA expression levels in the TCGA cohort when stage II/III patients were limited to those with histological grade 3. There was no significant difference in OS between the *LGR5* high and low groups (log-rank test, *P* = 0.7198). Univariate and multivariate analyses for prognostic factors were performed using the Cox proportional hazard regression model. However, there was no significant difference in the univariate analysis of the TCGA cohort (HR = 1.08; 95% CI: 0.70–1.67; *P* = 0.72).

## Discussion

*LGR5* is an independent prognostic factor in PD-AC stages II and III. Although PD-AC has a poor prognosis [9], the related factors are not well understood. PD-AC exists as solid and non-solid subtypes. The prognosis of patients with the non-solid subtype with fibrosis is poor [12]. In our study, most PD-AC cases were non-solid, but no clear difference was observed in the prognosis of both non-solid and solid subtypes. *LGR5* is also a promising gastric CSC marker, and high *LGR5* expression in the poor prognosis group may suggest an involvement of CSCs in determining the prognosis. Therefore, *LGR5* may be a therapeutic target in PD-AC and improve PD-AC patient prognoses.

Migration ability and epithelial-mesenchymal transition (EMT) are increased in poorly differentiated gastric cancer [13]. Therefore, the poor prognosis of patients with high *LGR5* expression may be related to the histological features of poorly differentiated cancer represented by enhanced migration ability, EMT-related protein expression, and *LGR5* expression. Cancer cell migration is known to affect prognosis in gastric cancer [13]. Additionally, *LGR5* expression, although not in the stomach, is related to migration ability and EMT [14]. In our study, the correlation between vascular invasion and high *LGR5* expression may support an association of *LGR5* expression with EMT. High *LGR5* expression and EMT were reported to be correlated in gastric cancer [15, 16]. Furthermore, Zhang et al. reported that the LGR5 ligand RSPO2 promotes EMT in gastric cancer cells by activating WNT/β-catenin signaling via *LGR5* [17]. Therefore, elucidation of the relationship between *LGR5* and *RSPO2* in PD-AC may lead to the development of new therapeutic methods and an improved prognosis for PD-AC.

*LGR5* expression in the tumors of various organs has been widely investigated, mainly using immunostaining. Although some reports have indicated that high *LGR5* expression is associated with a poor prognosis [18, 19], others have used RNAscope, which is considered to be more reliable [20, 21], and have reported that high *LGR5* expression correlates with a good prognosis specifically in pancreatic ductal adenocarcinoma [21]. We previously utilized RNAscope and showed that high *LGR5* expression might be a poor prognostic factor in breast cancer [22].

Several reports have investigated *LGR5* expression in gastric cancer. One RNAscope-based study did not uncover any difference in the OS of gastric cancer patients with varying expression of *LGR5* [23]. In other study of gastric cancer, high immunostaining scores for *LGR5* were significantly associated with an increased risk of death [24]. Taken together, these data indicate that the role of *LGR5* may be tissue-specific, and that its protein and mRNA regulation may be more complex than it seems.

While in our study, higher levels of *LGR5* expression were associated with adverse prognosis, no significant differences in the overall survival were detected in TCGA cohort. This discrepancy might be due to ethnic or technical differences.

In addition, Bu et al. reported that *LGR5* expression is associated with a favorable prognosis, although this was limited to stages I and II [25]. Furthermore, *LGR5* expression was associated with a high stage and lymph node metastasis [23]. Xi et al. reported that high *LGR5* expression is associated with poorly differentiated cancer [26]. However, Bu et al. reported that *LGR5* is highly expressed in well-differentiated cancer [25]. Additionally, in studies using RNAscope, high *LGR5* expression is correlated with well-differentiated cancer [27]. However, there is no comparison between *LGR5* expression and prognosis in PD-AC using RNAscope, and the association between high *LGR5* expression and poor prognosis in poorly differentiated cancer is a new finding.

The tendency for low *LGR5* expression in EBV-positive tumors may be a feature of EBV-associated gastric cancer. According to a novel molecular pathological classification [28], EBV-associated gastric cancer is recognized as a distinct type of gastric cancer with a good prognosis [29]. The Cancer Genome Atlas Research Network reports that gastric cancer is divided into four types [28]. Therefore, to accumulate further knowledge in the future, it is necessary to analyze *LGR5* expression in each type.

Our study has some limitations. This study included a relatively small sample size, which may have led to unreliable estimates. *LGR5* expression and migration must be investigated in cultured cells; additionally, *LGR5* expression must be analyzed in EBV-infected cells.

## Conclusions

The association of *LGR5* expression and patient prognosis in poorly differentiated gastric cancer may be applicable to the development of LGR5 targeted therapy and prognostic markers, but further study is desired.

## Data Availability

All data generated and analyzed during the current study are available from the corresponding author on reasonable request.

## Abbreviations

CSC: Cancer stem cell
LGR5: Leucine-rich repeat-containing G-protein-coupled receptor 5
PD-AC: Poorly differentiated adenocarcinoma
TMA: Tissue microarray
TILs: Tumor-infiltrating lymphocytes
OS: Overall survival
EMT: Epithelial-mesenchymal transition
